# Three‐dimensional stratification pattern in an old‐growth lowland forest: How does height in canopy and season influence temperate bat activity?

**DOI:** 10.1002/ece3.8363

**Published:** 2021-11-21

**Authors:** Maude Erasmy, Christoph Leuschner, Niko Balkenhol, Markus Dietz

**Affiliations:** ^1^ Plant Ecology and Ecosystems Research Albrecht‐von‐Haller Institute for Plant Sciences University of Goettingen Goettingen Germany; ^2^ Wildlife Sciences Faculty of Forest Sciences University of Goettingen Goettingen Germany; ^3^ Institute for Animal Ecology and Nature Education Laubach Germany

**Keywords:** Bialowieza forest, gaps, insectivorous bats, seasonality, three‐dimensional habitat use

## Abstract

The study of animal–habitat interactions is of primary importance for the formulation of conservation recommendations. Flying, gliding, and climbing animals have the ability to exploit their habitat in a three‐dimensional way, and the vertical canopy structure in forests plays an essential role for habitat suitability. Forest bats as flying mammals may seasonally shift their microhabitat use due to differing energy demands or changing prey availability, but the patterns are not well understood. We investigated three‐dimensional and seasonal habitat use by insectivorous bats in a temperate lowland old‐growth forest, the *Belovezhskaya Pushcha* in Belarus. We acoustically sampled broadleaved and mixed coniferous plots in the forest interior and in gaps in three heights during two reproductive periods (pregnancy/lactation vs. postlactation). In canopy gaps, vertical stratification in bat activity was less pronounced than in the forest interior. Vertical activity patterns differed among species. The upper canopy levels were important foraging habitats for the open‐space forager guild and for some edge‐space foragers like the Barbastelle bat *Barbastella barbastellus* and the soprano pipistrelle *Pipistrellus pygmaeus*. *Myotis* species had highest activity levels near the ground in forest gaps. Moreover, we found species‐dependent seasonal microhabitat shifts. Generally, all species and species groups considered except *Myotis* species showed higher activity levels during postlactation. *Myotis* species tended toward higher activity in the forest interior during postlactation. *P*. *pygmaeus* switched from high activity levels in the upper canopy during pregnancy and lactation to high activity levels near the ground during postlactation. We conclude that a full comprehension of forest bat habitat use is only possible when height in canopy and seasonal patterns are considered.

## Graphical abstract

We investigated three‐dimensional and seasonal habitat use by insectivorous bats in a temperate lowland old‐growth forest. We identified guild‐ and species‐specific differences in canopy height use and species‐dependent seasonal microhabitat shifts that can be explained by ecomorphological adaptations.

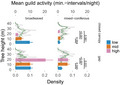

## 1 INTRODUCTION

Forests are three‐dimensionally structured ecosystems, where plant species and resources are heterogeneously distributed in time and space (Muscolo et al., 2014; Perry et al., 2018). Knowledge about how this three‐dimensional heterogeneity impacts the spatio‐temporal behavior of forest animals is essential for the formulation of conservation measures (e.g., Alder et al., 2020; Charbonnier et al., 2014; Ruczynski & Barton, 2020). The forest canopy as the upper layer of vegetation formed by tree crowns is a particularly important habitat and resource element used by vertebrate and nonvertebrate forest animals (Lowman et al., 2013; Nakamura et al., 2017).

A few decades ago, forest research was restricted to ground‐based methods due to technical limitations and inferences on species interactions and population dynamics within the canopy were mainly deduced from ground observations (Lowman et al., 2013; Nakamura et al., 2017). The development of new technologies such as canopy access facilities (e.g., cranes) and remote sensing systems (e.g., drones) led to an increasing accessibility of forest canopies (Basset et al., 2003; Froidevaux, 2016; Jung et al., 2012; Lowman et al., 2012, 2013; Nakamura et al., 2017; Ozanne et al., 2021; Unterseher et al., 2007). Vertebrates using the forest canopy for moving, feeding, or resting actively choose their microhabitats by vertically switching between forest layers. This has been shown for birds (Acharya & Vijayan, 2017; Jayson & Mathew, 2003; Pearson, 1971; Rajaonarivelo et al., 2020; Shaw et al., 2002; Walther, 2002), gliding squirrels (Krishna et al., 2016), or monkeys (Enstam & Isbell, 2004; Li, 2007; Pinheiro et al., 2013) in different climatic regions. Bats as three‐dimensionally moving organisms can exploit the forest canopy, the free space above the canopy and the interstrata free space. Studies on the role of tree crowns for bats originated in the tropics, where a number of studies revealed vertical stratification of various diversity metrics caused by species‐inherent ecomorphological constraints and specializations in foraging behavior (Bernard, 2001; Carvalho et al., 2013; Duya et al., 2017; Fraixedas Nunez et al., 2019; Gregorin et al., 2017; Henry et al., 2004; Kalko & Handley, 2001; Ramos Pereira et al., 2010; Rex et al., 2011; Silva et al., 2020; Tiago Marques et al., 2016). Such height stratification patterns were also found for arthropods across climatic regions (Ashton et al., 2016; Basset et al., 2015; Oguri et al., 2014; Stork & Grimbacher, 2006). In temperate regions worldwide, studies focusing on the three‐dimensional space use in forest bats have not yet revealed consistent height patterns (United States: Hayes and Gruver (2000), Menzel et al. (2005), Kennedy et al. (2014), Australia: Adams et al. (2009), New Zealand: Scrimgeour et al. (2013)). In Europe, Froidevaux et al. (2014) did not detect any layer preferences (ground vs. canopy) within guilds. Plank et al. (2012) found species‐dependent activity differences between strata and according to Collins and Jones (2009) and Mueller et al. (2013), species or species group activities and species assemblages differed between canopy layers.

In the forest interior, forest bat activity is more strongly confined to certain heights than at forest edges such as forest tracks or water bodies (Adams et al., 2009; Tiago Marques et al., 2016). In the absence of vegetation clutter, the flight heights used by bats are not determined by physical constraints but are rather depending on species‐dependent prey preferences. Indeed, bats’ choice of adequate foraging habitats results from an interaction of prey species and their abundances (Andreas et al., 2012a; Ferreira et al., 2017; Salvarina et al., 2018), current energy requirements (Lucan & Radil, 2010; Ruczynski et al., 2017; Russ et al., 2003), and local competitive interactions (Andreas et al., 2012b; Roeleke et al., 2018; Vasko et al., 2020). Differing seasonal habitat requirements can thus be reflected in seasonal height use shifts, as Staton and Poulton (2012) and Plank et al. (2012) showed for temperate bats. Seasonal shifts in bat activity have been shown furthermore to occur between habitats (Ferreira et al., 2017; Heim et al., 2016; Kelm et al., 2014; Lucan & Radil, 2010; Roeleke et al., 2018; Russ et al., 2003; Vasko et al., 2020).

We acoustically sampled the vertical height use of a temperate forest bat assemblage in a European lowland old‐growth forest. We compared the activity of guilds, the activity of dominant species, and species community composition in two forest habitats for the ground, mid, and high canopy layer in the forest interior and in canopy gaps for the two time periods pregnancy/parturition and lactation/postlactation. This way, we were able to assess seasonal preferences in three dimensions both locally (vertically in the forest interior and adjacent gaps) and at a broader spatial scale (broadleaved vs. mixed coniferous forests).

Specifically, we hypothesized that

1. stratification of bat activity is more accentuated in the forest interior than in forest gaps
2. forest layers are differently used by guilds and species depending on their ecomorphology and prey preferences
3. forest layers are differently used by guilds and species depending on the reproductive season.

## 2 MATERIAL AND METHODS

### 2.1 Study area

The study was conducted in the “Belovezhskaya Pushcha” National Park (*BPNP*) in Western Belarus. The National Park is largely dominated by temperate and hemiboreal woodlands (approximately 80% of the total 153,000 ha, Nikiforov and Bambiza (2008)) at elevations of 134–202 m a.s.l. and is part of the *Belovezhskaya Pushcha*/ *Puszcza Białowieska* (*BP*) forest complex which extends beyond the Polish–Belarusian border (Jaroszewicz et al., 2019). The climate is subcontinental, with a mean annual air temperature of 7.3°C and an average annual precipitation of 625 mm (period 1985–2015, Boczoń et al. (2018)). Mixed coniferous forests are the prevailing vegetation, reflecting the transitional character of *BP* between nemoral broadleaved and boreal coniferous forests (Nikiforov & Bambiza, 2008). *Pinus sylvestris* L. (Scots pine) is the dominating tree species in more than half of the forest stands on the Belarusian side of *BP* (Falinski, 1986; Nikiforov & Bambiza, 2008). Besides *Alnus glutinosa* L. (common alder) in swamp forests, English oak (*Quercus robur* L.), European hornbeam (*Carpinus betulus* L.), and small‐leaved lime (*Tilia cordata* Mill.) form broadleaved mixed forests on sites not influenced by groundwater. In almost all forest stands in *BP*, *Picea abies* (L.) H. Karst. (Norway spruce) is present as an admixture (Falinski, 1986).

Our study plots were located within the “strict reserve” of the National Park, where management activities are prohibited on an area of 57,000 ha. To guarantee independent sampling of bats as flying mammals, all plots were located more than 7 km from each other. All plots were at least 1 km away from the nearest settlement to avoid anthropogenic influences and at least 300 m from external forest borders to minimize edge effects. Furthermore, plots were located more than 1 km distant to water bodies or courses to reduce the influence of water on bat activity (Fukui et al., 2006; Grindal, 1998; Salvarina et al., 2018; Vindigni et al., 2009).

Our study design covered two different habitat types for comparison. Four plots were located in mixed Pino‐Quercetum stands (mixed coniferous forest), which represent the dominating forest community on the Belarusian side of *BP* (Falinski, 1986). These forest stands are dominated by *P. sylvestris* with varying admixtures of *P*. *abies* in the upper canopy and *P*. *abies* and *Q*. *robur* in the second tree layer. The understory was dominated by young *Picea* trees which create rather dense inner stands with respect to available flight space. Four plots were located in broadleaved Tilio‐Carpinetum stands, a mesotrophic forest community with frequently *Q*. *robur* and more rarely *Tilia cordata* or *Acer platanoides* dominating the uppermost canopy layer, and a rather dense subcanopy created mainly by *C*. *betulus* and *P*. *abies*. The forest interior was less dense compared to mixed coniferous stands due to lower stem densities. Each plot consisted of two subplots, with an average distance of 154 ± 85 m from each other. One subplot was located in the forest interior, and the other in an adjacent forest gap. All gap plots had been created by fallen trees and were located within the forest matrix, without connections to other open structures.

### 2.2 Bat sampling

We used acoustic recording techniques to estimate bat activity. Devices automatically recording ultrasound were deployed at the plots (batcorder 3.0, EcoObs GmbH Nuremberg). We used the recording mode “Auto‐Timer” and the following recording settings: quality = 20, threshold = −27 dB, post‐trigger = 400 ms, critical frequency = 16 kHz. Recordings automatically ran from sunset until 1 h after sunrise. Following recommendations from Weller and Zabel (2002) and Britzke et al. (2013), omnidirectional ultrasonic microphones were slightly inclined upward and the space surrounding them was void of vegetation clutter to minimize detection probability bias.

In each subplot, batcorders were installed at three heights to collect a three‐dimensional acoustic image of bat activity along a vertical gradient in the plot center. In the forest interior, batcorders were placed at the plot center (see 2.4 Stand structural data). In gaps, batcorders were placed in the subjective gap center. A rope‐and‐pulley system was used to suspend the batcorders. With a slingshot, we shot an auxiliary rope into a suitable branch fork. By means of this auxiliary rope, we pulled up the final string to which batcorders were attached at three heights. If no adequate tree was present in the gap center, we shot auxiliary ropes in two suitable trees on each side of the gap. This way, a rope was stretching from one side of the gap to the other side at canopy height. In the gap center, batcorders were attached to this rope using a vertically hanging line. We anchored the line in this central position with side ropes and tent pegs.

**FIGURE 1:**
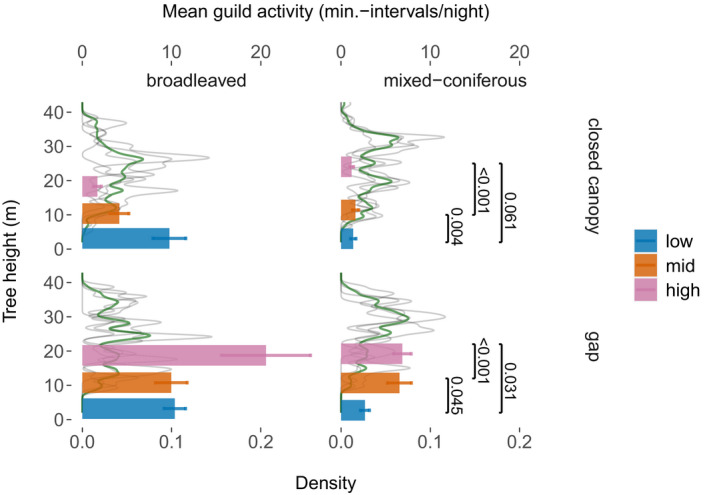
Density function of tree height profiles in individual subplots (light gray, *n* = 4) and averaged over all plots (thick green line). Bars show the mean nighttime activity (± standard error) in minute‐intervals/night for total bat guild plotted at the mean batcorder heights. Significant *p*‐values of post hoc Tukey tests adjusted for multiple comparisons for the three height levels for both habitats combined from the GLMM fitting total bat activity

We investigated bat activity in three heights within each subplot. Ground sampling (*low* stratum, space dominated by tree stems, and understory vegetation) was established at an average height of 3 m (SD = 0.7 m, min = 1.6 m, max = 4.2 m). Midcanopy sampling (*mid* stratum, space of subcanopy trees) was conducted at 11 m (SD = 1.2 m, min = 9 m, max = 12.8 m) and high‐canopy (*high* stratum, space between subcanopy and canopy trees) sampling at 19 m (SD = 3.7 m, min = 13.1 m, max = 26.6 m). We tried to evenly spread the three batcorders over the height spectrum. However, we were not able to position the highest batcorder at the crown‐top level of the tallest trees (Figures 1 and A1).

The batcorder array was set up simultaneously in a gap subplot and the neighboring forest interior subplot. For technical reasons, the number of recording nights per batcorder differed between 3 and 12 nights (mean 8 recording nights, SD = 2.5). Sampling took place on 72 nights between 31 May and 4 September 2015. To catch seasonal effects on bat activity, we considered two time periods with similar sampling effort for each habitat and canopy structure (period I: 192 samples, period II: 196 samples; Table 1). However, while period I included samples from four plots (accordingly eight subplots) per habitat, in period II only 3 plots (corresponding to six subplots) per habitat were sampled (Table 1). Due to technical problems, the highest batcorder in subplot E2 in broadleaved gaps was not working in period I; however, all synchronously recorded sequences in the other heights were included in the analyses, since the models used allow for differing sample sizes. Period I until July 3rd included gestation, parturition, and lactation of the offspring, while period II encompassed weaning of the young and the beginning of their independent flights (Table A1). Temperature was measured internally in each batcorder and stored every 15 min (Figure A2). On ten nights, precipitation events of low impact took place (0.3–5.5 mm per night, measured between 18:00 and 6:00). All precipitation nights were included in the analyses, since exploratory analyses did not show any influence of these rare and low‐intensity events on bat activity. Every height stratum in each habitat and canopy structure type was sampled between 29 and 37 nights during our field campaign.

**TABLE 1:** Fitted effects for total bat activity from a generalized linear mixed model with negative‐binomial distribution assumed (*n* = 388)

Predictors	IRR ± SE	Stat.	*p*
habitat [mixed coniferous]	0.35 ± 0.15	−2.42	.**015**
structure [gap]	2.09 ± 0.93	1.66	.098
height [mid]	0.55 ± 0.10	−3.25	.**001**
height [high]	0.34 ± 0.07	−5.39	**<.001**
mean night‐time temperature	1.11 ± 0.03	4.29	**<.001**
structure [gap] * height [mid]	2.18 ± 0.53	3.22	.**001**
structure [gap] * height [high]	5.40 ± 1.39	6.52	**<.001**

Random effects: subplot = 16 levels, date = 72 levels. IRR = incidence rate ratio, SE = standard error. Habitat fitted against broadleaved, canopy structure “gap” against the forest interior, and heights against the ground layer.

Significance threshold *p* < .05 in bold.

### 2.3 Acoustic data analysis

We collected acoustic bat calls and used these recordings to identify bat species. The software batIdent (EcoOb GmbH) automatically identifies species and assigns identification probabilities. López‐Baucells et al. (2019) found that a combination of automatic and manual methods is effective in identifying bat calls. Hence, we used a combination of automatic bat call identification and manual postvalidation of these assignments using the software bcAnalyze2 (EcoOb GmbH). Parameters and literature used for manual species identification are given in Erasmy et al. (2021). Bat calls not identified to species were combined into sonotypes. Recently, 16 bat species have been described for the Belarusian side of BP (Dietz et al., 2018). We used the *pipistrellus* sonotype for calls from unidentified *P*. *pipistrellus* and *P*. *pygmaeus*, *myotis* sonotype for unidentified calls from *M*. *alcathoe*, *M*. *brandtii*, and *M*. *daubentonii*, and *nyctaloid* sonotype for unidentified calls from *E*. *nilssonii*, *E*. *serotinus*, *N*. *noctula*, *N. leisleri*, and *V*. *murinus*. A few calls were attributed to *Plecotus* spec. These calls most probably refer to *Plecotus auritus*, since *P*. *austriacus* has only rarely been recorded in *BP* (Sachanowicz et al., 2006). We performed a first set of statistical analyses on bat guild level and used the guild attribution of bat species following Erasmy et al. (2021) and Mueller et al. (2012). Edge‐space foragers (*ESF*) included *B*. *barbastellus*, *P*. *pipistrellus*, *P*. *pygmaeus*, *M*. *brandtii*, *M*. *daubentonii*, *M*. *alcathoe* and not further specified *Myotis* spec., narrow‐space foragers (*NSF*) comprised *Plecotus auritu*s and *M. nattereri*, and open‐space foragers (*OSF*) *P. nathusii*, *N*. *leisleri*, *N*. *noctula*, *E*. *nilssonii*, *E*. *serotinus*, *V. murinus*, and all not further specified nyctaloid calls.

When analyzing bat recordings, we cannot distinguish between one individual recorded several times and several individuals recorded once. For this reason, Hayes (1997) and Kalcounis et al. (1999) proposed the use of an activity index instead of the number of recorded sequences as a method to take account of this issue. We used the number of 1‐minute intervals with bat calls per night as an activity index [see Mueller et al. (2012) for a similar methodology and Erasmy et al. (2021) for a detailed description of this index]. This index evens out the effects of very high activity levels produced by species hunting in front of the microphone or by species with intercall intervals exceeding the post‐trigger time (e.g., *N. noctula*).

Individual bat species differ in echolocation call intensities. This induces varying interspecies detection probabilities in the same habitat and under identical weather conditions (Britzke et al., 2013). We therefore refrained from comparing activity patterns between guilds or species. Detection probabilities within species vary with vegetation clutter and weather conditions (Bender et al., 2015; Britzke et al., 2013; Gorresen et al., 2008; Yates & Muzika, 2006). All batcorders in the forest interior were surrounded by vegetation‐free space to create similar recording situations and to minimize attenuation effects on bat calls through leaves and branches. We sampled the same habitat type at multiple plots with differing vegetation structures surrounding our batcorders. Since we were interested in habitat effects on bat activity, we are confident that these differing forest structural patterns from within the same habitat are suited to account for detection probability differences due to vegetation clutter.

Our batcorder array sampling synchronously at three heights possessed a pitfall: Since every microphone was recording on a single device, high‐intensity bat calls were likely to reach the neighboring batcorder microphone and thus trigger the same activity recording in adjacent batcorders. We therefore manually checked all recordings, identified calls with the same timestamp from the same species/sonotype at neighboring batcorders, and assigned them to the batcorder with the strongest signal (Tiago Marques et al., 2016).

### 2.4 Stand structural data

**FIGURE 2:**
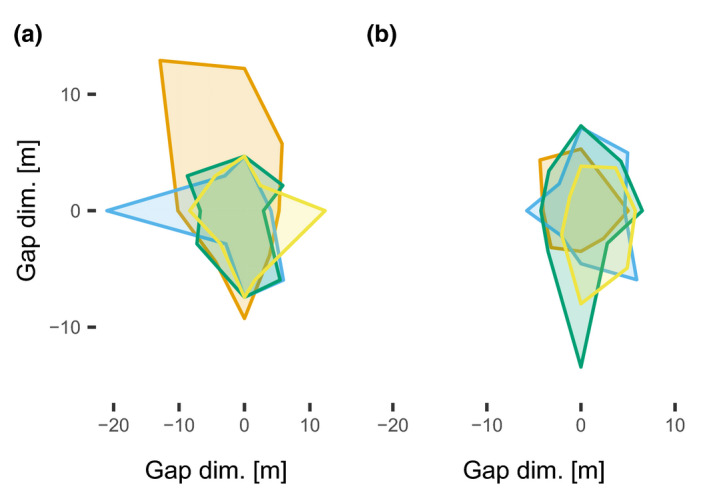
(a) and (b) Projected gap areas of the coniferous (*n* = 4) and broadleaved (*n* = 4) forest sites in a projected coordinate system with 0 as the plot center where the batcorder was placed and gap dimensions showing the extent of each gap site in differently colored shades

In gaps, we estimated gap area following Runkle (1982) by determining the edge of crowns in eight directions from the gap center. Gap sizes ranged from 56 to 265 m^2^, with an average gap size in broadleaved plots of 78 ± 23 m^2^ and an average gap size of 156 ± 77 m^2^ in mixed coniferous (Figure 2).

Plots in the forest interior were established on an area of 1000 m^2^ (17.8 m radius around the batcorder as the plot center). We measured the height of 12–18 trees per plot and used the nonlinear regression equation reported by Petterson (1955) to predict the height of all trees not measured. These height measures were used for depicting the plot height profiles of trees within the plot (forest interior subplots) or surrounding the gap (forest gap subplots) for the two habitat types considered (Figure 1).

### 2.5 Statistical analyses

All statistical analyses were performed within the R 4.0.3 software environment (R Development Core Team, 2019). To test for the influence of structural and seasonal effects on bat activity, separate models were fitted for total bat activity, for the activity of each bat guild (*OSF*, *ESF*, and *NSF*) and of each of the dominant species within each guild with the 1‐min activity index as a response variable. To disentangle species‐dependent activity differences within the *ESF* guild, the activities of the main *ESF* species (Barbastelle bat, soprano pipistrelle, and *Myoti*s spec.) were separately fitted. *Myotis brandtii* (7% of *ESF* activity) and not further identified *Myotis* spec. (20% of *ESF* activity) were first analyzed in separate analyses. The patterns identified were qualitatively identical, and their activity data were jointly analyzed as *Myotis* spec. to increase sample size. We fitted linear mixed models using the package glmmTMB (Brooks et al., 2017) and validated model assumptions with the DHARMa package (Hartig, 2020). We accounted for seasonal variations in bat activity by monitoring throughout the summer and by integrating Julian date as a random factor (*n* = 72, total bat activity model) or two recording periods corresponding to pregnancy/lactation and postlactation period as fixed effect into our models (all other models; Hayes, 1997; Skalak et al., 2012; Vasko et al., 2020). Subplot (*n* = 16) was added as a random factor to account for subplot‐dependent variation not captured by the predictors used. A set of candidate models including all two‐way interactions between height, canopy openness (gap vs. forest interior), season (pregnancy/lactation vs. postlactation), and forest habitat (broadleaved vs. mixed coniferous) were fitted for each guild/species with assumed negative‐binomial distributions. Mean nighttime temperature was added as a simple predictor, since several studies identified temperature as an important predictor both for bat and insect activity (e.g., Dajoz, 2000; Froidevaux et al., 2021; Mueller et al., 2012; Wolbert et al., 2014). Post hoc testing for effects with more than two levels was done using Tukey’s honestly significant difference test with a correction factor for multiple comparisons using the pairs function within the emmeans package (Lenth, 2020). The best fitting and most parsimonious model from this candidate set was identified using Akaike’s information criterion adapted for small sample sizes (AICc) and chosen within AICc values below 2 (Brewer et al., 2016; Burnham & Anderson, 2004). All candidate models including their differences in AICc values are shown in Table A2. Moreover, we calculated marginal *R* ^2^‐values for the best fitting model using the Nakagawa equation (Table A2; Nakagawa and Schielzeth (2013), Lüdecke et al. (2021)). The best model is presented using restricted maximum likelihood (REML). Predictions used for plotting were calculated using the emmeans package (Lenth, 2020).

We applied nonmetric multidimensional scaling based on the Bray–Curtis similarity metric on species activity data to describe species assemblages (function metaMDS from the R package ‘vegan’, Oksanen et al. (2020). This function was applied to the activity data of all species/species groups present with more than 20 min‐intervals in the field campaign. *V. murinus*, *Plecotus spec*., *M. alcathoe*, *M. dasycneme*, and *E. serotinus* were excluded from this multivariate analysis due to their rare observation.

## 3 RESULTS

### 3.1 General patterns of bat activity

During 72 measuring nights, we recorded a total of 4316 bat call sequences (transformed into 2507 min‐intervals per night). During 90 of the 388 batcorder sessions, no bat calls were recorded. The recordings were assigned to the three guilds *OSF*, *ES*F, and *NSF*, with 72% of the total activity belonging to *ESF* species, 24% to *OSF* and 4% to *NSF* species. 64.5% of the activity data could be assigned to one of the 13 species identified, and the remaining sequences were attributed to species groups or sonotypes (see Section 2.3). 73% of the total activity observed in the study was recorded in broadleaved forests and 27% in mixed coniferous forests. About a quarter of total activity (27%) was observed in the forest interior and 73% in gaps. Bat total activity was evenly distributed over all three heights (high: 37%, mid: 29%, ground: 33%; Table A3 summarizes the raw data).

Total bat activity revealed opposing height patterns between forest gaps and the forest interior. In gaps, the highest activity was recorded in the upper canopy, and activity levels were lower with decreasing heights (Table 1). In the forest interior, however, the highest activity levels were recorded at the ground, with lower activity levels higher in the canopy (Table 1; Figure 1).

**FIGURE 3:**
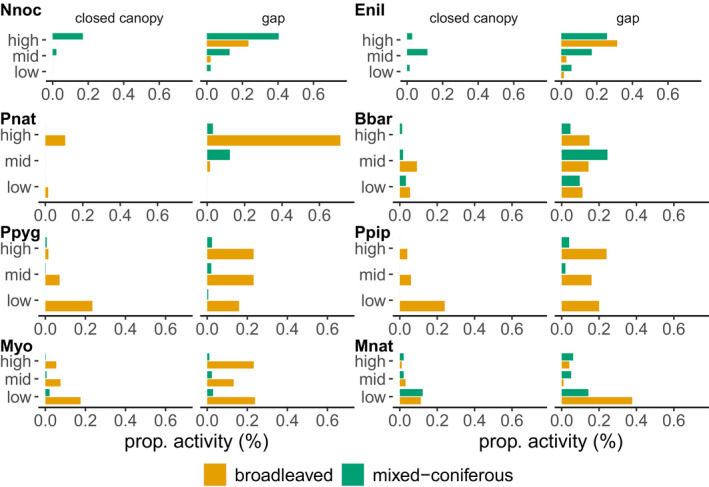
Proportional species activity spent in the habitat structures considered relative to the total activity of this species in minute‐intervals/night for dominant species. Nnoc = *Nyctalus noctula*, Enil = *Eptesicus nilssonii*, Pnat = *Pipistrellus nathusii*, Bbar = *Barbastella barbastellus*, Ppyg = *Pipistrellus pygmaeus*, Ppip =* P*. *pipistrellus*, Myo = *Myotis*. *spec*. (all *Myotis* spec. except *M. nattereri*), Mnat = *M. nattereri*

None of the species identified was recorded exclusively in either habitat, canopy structure, or height. However, differences in the proportional activity spent in each microhabitat became evident on species level (Figure 3). *N. noctula* was detected proportionally more often in gaps (81% of the total *N. noctula* activity) and in the upper canopy (81%) in mixed coniferous habitats (74%; Figure 3). *P. nathusii* bats spent almost their total activity in gaps (88%) and in the upper canopy (85%) in broadleaved forests (85%; Figure 3). *B*. *barbastellus* was equally active in broadleaved and mixed coniferous habitats but spent more time in gaps (79% of activity) with half of its activity at the midcanopy layer (50%; Figure 3). *P*. *pygmaeus* bats generally spent most of their activity in gaps (67%) distributed equally over all three heights (Figure 3). In the forest interior, they were most active at the ground (71% activity spent; Figure 3). 94% of their total activity was spent in broadleaved forests. *P*. *pipistrellus* showed a proportional time activity pattern similar to *P*. *pygmaeus* (Figure 3). *Myotis* spec. spent most of their activity in broadleaved forests (91%) and were detected slightly more often in forest gaps (67%) and at the ground (46%; Figure 3). *M*. *nattereri* foraged in both habitat types, especially at the ground level (76%) and in forest gaps (68%; Figure 3).

### 3.2 Guild‐dependent activity stratification

**FIGURE 4:**
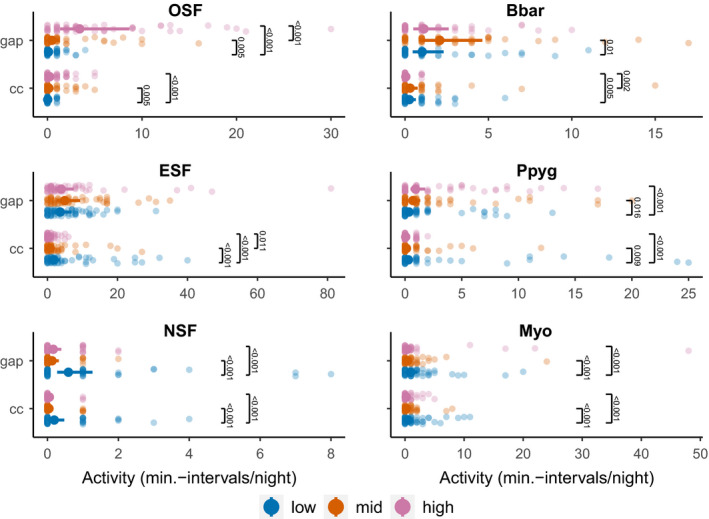
Estimated marginal means from the GLMMs for bat guild and species activity with 95% confidence levels as error bars. Raw activity data are plotted as transparent points in the background. For *OSF*s, the highest data point (69 min‐intervals/night) was excluded from the plot for a better visualization. Significances of contrasts were corrected using Tukey’s post hoc test for multiple comparisons. *OSF*: open‐space foragers, *NSF*: narrow‐space forager, *ESF*: edge‐space forager, Myo: *Myotis* spec. (*Myotis brandtii* and undefined *Myotis* spec. combined), Bbar: *B*. *barbastellus*, Ppyg: *P*. *pygmaeus*. Colored bars indicate the three batcorder heights low (blue), mid (orange), and high (rose)

Calls belonging to *OSF*s were recorded in 36% of sample nights. The best model fitting *OSF* activity revealed a differing height activity pattern between canopy gaps and the forest interior (Table 2): In gaps, *OSF*s were significantly more active in the upper canopy stratum, whereas their predicted activity levels for midcanopy heights and the ground were negligible (Figure 4). In the forest interior, however, both mid‐ and high‐canopy heights revealed significantly higher *OSF* activity levels compared to the ground batcorder (Figure 4). *OSFs* were equally active in broadleaved and mixed coniferous forests, and temperature was significantly and positively influencing *OS*F activity levels (Table 2). Most calls from the *OSF* guild were unidentified nyctaloid calls (60% of all *OSF* calls). *E*. *nilssonii* and *P. nathusii* accounted for 12% and 11%, respectively, of *OSF* calls. Species models for this guild were not fitted due to a low number of observations for single species.

**TABLE 2:** Fitted effects of forest structure parameters on the activity of bat guilds and dominant species/species groups

	Height [mid]	Height [high]	Structure [gap]	Habitat [C]	Period [II]	Temp	Height [mid] * period [II]	Height [high] * period [II]	Height [mid] * structure [gap]	Height [high] * structure [gap]	Structure [gap] * period [II]	Structure [gap] * height [mid]	Structure [gap] * height [high]
*OSF*													
IRR ± SE	7.47 ± 6.34	29.31 ± 23.62	2.76 ± 2.45	3.28 ± 2.32	4.9 ± 3.89	1.13 ± 0.03	0.55 ± 0.45	0.09 ± 0.07	0.73 ± 0.38	3.42 ± 1.76	2.38 ± 0.91		
Stat.	2.37	4.19	1.14	1.68	2	4.17	−0.73	−3.18	−0.6	2.38	2.27		
*p*	.**018**	**<.001**	.254	.094	.**045**	**<.001**	.464	.**001**	.549	.**017**	.**023**		
*ESF*													
IRR ± SE	0.44 ± 0.12	0.32 ± 0.09	2.02 ± 0.95	0.2 ± 0.09	1.52 ± 0.31	1.06 ± 0.02	1 ± 0.27	0.43 ± 0.12	3.07 ± 0.85	5.21 ± 1.59			
Stat.	−3.04	−3.96	1.5	−3.53	2.05	2.54	0.01	−2.99	4.07	5.39			
*p*	.**002**	**<.001**	.135	**<.001**	.**041**	.**011**	.992	.**003**	**<.001**	**<.001**			
*NSF*													
IRR ± SE	0.22 ± 0.07	0.28 ± 0.09	6.75 ± 4.59	0.66 ± 0.37	3.23 ± 1.67	1.03 ± 0.05					0.21 ± 0.13		
Stat.	−4.5	−4.09	2.81	−0.73	2.27	0.61					−2.61		
*p*	**<.001**	**<.001**	.**005**	.466	.**023**	.541					.**009**		
*Bbar*													
IRR ± SE	1.17 ± 0.4	0.18 ± 0.1	3.99 ± 2.49	0.85 ± 0.49	1.6 ± 0.35	1.01 ± 0.03						1.72 ± 0.71	6.05 ± 3.69
Stat.	0.47	−3.14	2.23	−0.28	2.15	0.29						1.31	2.95
*p*	.636	.**002**	.**026**	.783	.**031**	.772						.19	.**003**
*Ppyg*													
IRR ± SE	0.64 0. ±0.26	0.45 ± 0.21	1.17 ± 0.59	0.05 ± 0.02	5.99 ± 1.82	1.11 ± 0.03	0.43 ± 0.17	0.19 ± 0.08	4.68 ± 1.69	13.34 ± 5.73			
Stat.	−1.08	−1.74	0.32	−6.37	5.9	3.45	−2.12	−4.05	4.28	6.03			
*p*	.279	.081	.746	**<.001**	<.001	.001	.034	**<.001**	**<.001**	**<.001**			
*Myotis*													
IRR ± SE	0.51 ± 0.15	0.67 ± 0.2	2.76 ± 1.57	0.11 ± 0.06	1.14 ± 0.42	1.11 ± 0.04	0.74 ± 0.31	0.33 ± 0.14			0.41 ± 0.16		
Stat.	−2.23	−1.37	1.79	−4.08	0.37	2.63	−0.72	−2.57			−2.27		
*p*	.**026**	.172	.074	**<.001**	.713	.**009**	.473	.**01**			.**023**		

Note

All generalized linear mixed models were fitted assuming negative‐binomial distributions. Random effects: subplot = 16 levels, 388 observations. Significant effects are shown in bold. IRR = incidence rate ratio, SE = standard error. Habitat mixed coniferous (C) compared against broadleaved, heights (mid, high) compared against ground, structure (canopy gaps against the forest interior), sampling period I against period II. Temp = mean nighttime temperature.

*ESF*s were active during 68% of all sample nights. The most parsimonious model showed a significantly differing height pattern between gaps and the forest interior (Table 2): In gaps, *ESF*s were equally active over the three heights considered (Table 2). In the forest interior, however, *ESF*s were most active at the ground (estimated marginal mean (*EMM*) activity from the model: 1.8 min‐intervals/night; Figure 4). Generally, broadleaved forests were significantly preferred by *ESF* bats (Table 2). Activity within this guild significantly increased with higher mean nighttime temperatures (Table 2). Height use in Barbastelle bats (23% of *ESF* activity) differed depending on the canopy structure. Barbastelle bats’ activity in canopy gaps was highest at mid heights (Figure 4). In the forest interior, Barbastelle bats were significantly more active at the ground and at mid heights compared to the highest layer. Barbastelle bats did not prefer any of the two habitat types, broadleaved or mixed coniferous (Table 2). In both habitat types, they were significantly more often recorded in gaps (Table 2). Temperature was not influencing Barbastelle activity (Table 2). Soprano pipistrelles made up 33% of the *ESF* activity. They showed an overall activity pattern similar to the one described for the *ES*F guild as a whole. The best fitting model showed a differing height activity pattern between gaps and the forest interior (Table 2). Soprano pipistrelles used the whole vertical canopy spectrum in canopy gaps, with significant higher activity levels in the highest layer (Figure 4). In the forest interior, however, their activity was restricted to the ground, with significant lower activity levels recorded both for the mid and high heights (Figure 4). Soprano pipistrelles preferred hunting in broadleaved forests; their activity levels in mixed coniferous forests were negligible (EMMs 0.056 ± 0.02 min‐intervals/night; Table 2; Figure 4). Mean nighttime temperatures had a positive influence on soprano pipistrelles’ activity levels (Table 2). *Myotis* species showed equal activity levels in canopy gaps and in the forest interior and they significantly preferred hunting in broadleaved forests (Table 2). Considering height segregation, *Myotis* species were most active at the ground (Figure 4). *Myotis* activity was increasing with increasing nighttime temperature (Table 2).

*NSF* bats were recorded in 18% of recording nights. They were significantly more active at the ground compared to mid‐ and high‐canopy layers and did not show any preference for a certain forest type (Figure 4). *NSF* activity levels in canopy gaps were higher than in the forest interior and were not influenced by mean nighttime temperatures (Table 2). *NSF*s were dominated by *Myotis nattereri*, with 89% of all *NSF* call sequences from this species.

**FIGURE 5:**
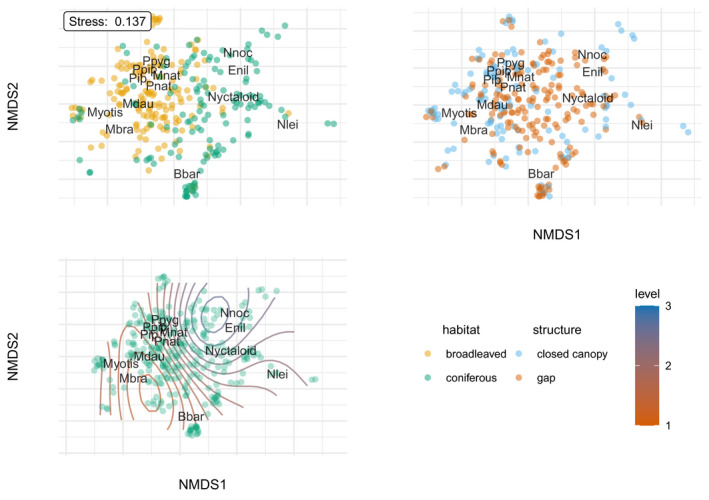
NMDS plots showing 2 of 3 dimensions for (a) habitat, (b) canopy structure, and (c) height levels depicted as isolines (levels 1–3). Bbar = *Barbastella barbastellus*, Enil = *Eptesicus nilssonii*, Mbra = *Myotis brandtii*, Mdau = *M*. *daubentonii*, Mnat = *M*. *nattereri*, Pip = *Pipistrellus* spec., Ppip =* P*. *pipistrellus*, Ppyg =* P*. *pygmaeus*, Nlei = *Nyctalus leisleri*, Nnoc = *N. noctula*

A clear pattern evident from the nonmetric multidimensional scaling was the species segregation between broadleaved and mixed coniferous plots, which mainly spread along the first NMDS axis explaining the greatest variance (Figure 5a; stress values of 0.14 with *k* = 3 dimensions and a maximum of 500 permutations starting from the previous best solution). The resulting linear fit (*R* ^2^) was 0.883. Nyctaloids were associated mainly with mixed coniferous plots, while *Myotis* and *Pipistrellus* species were more closely linked to broadleaved plots. The NMDS plot did not show a segregation between canopy structures (Figure 5b). Canopy height was depicted as a gradient in Figure 5c using a contour plot with isolines representing identical height levels. A clear transition from *N. noctula* and *E*. *nilssonii* over the nyctaloids (active at the highest canopy) to *M*. *brandtii* and *Myotis* spec. (most active at the ground) became apparent. Barbastelle bats, Pipistrelle bats, and Natterer’s bats were occupying intermediate height positions in ordination space (Figure 5c).

### 3.3 Seasonal activity pattern

**FIGURE 6:**
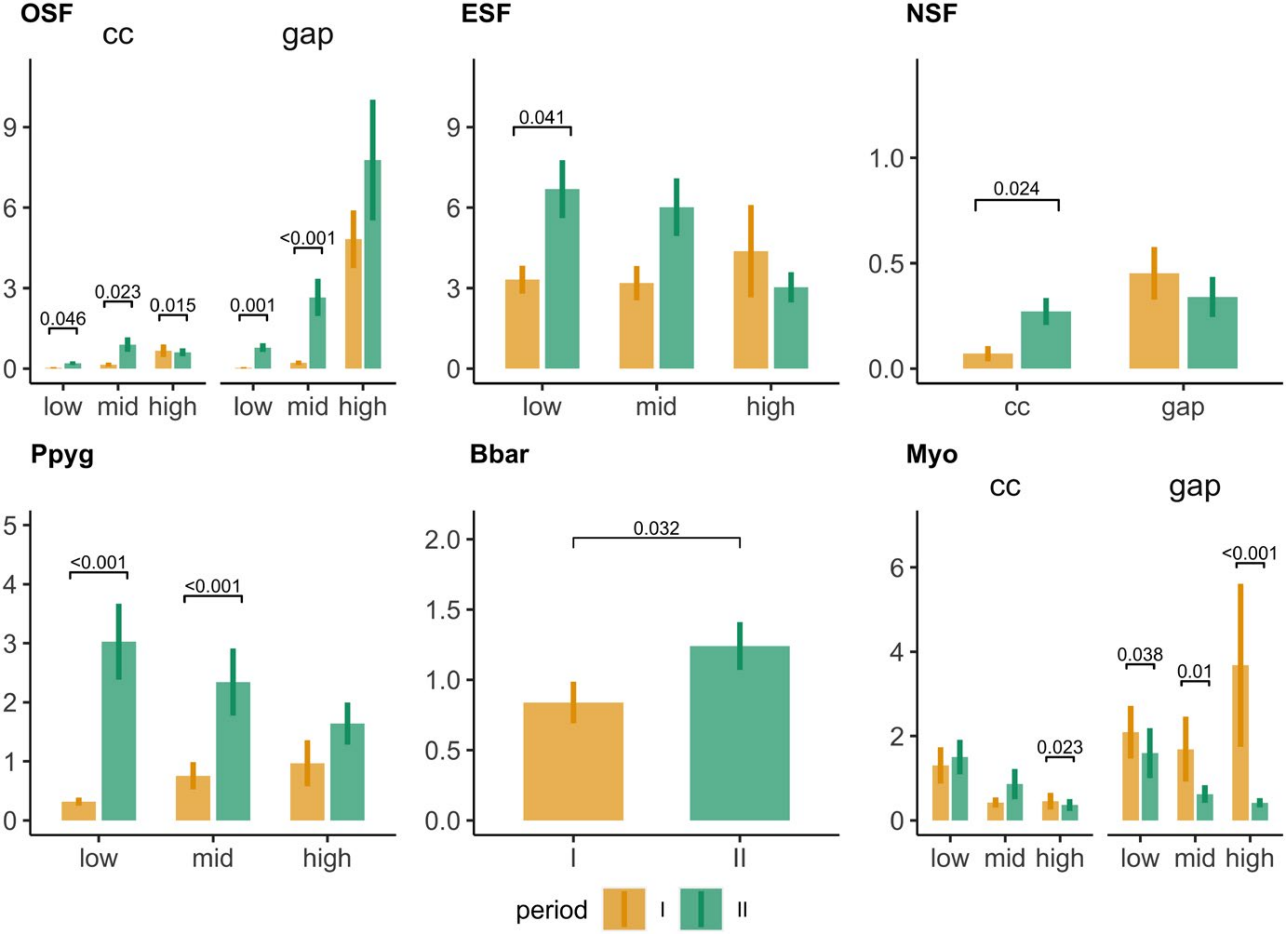
Seasonal mean nighttime activity changes in bat guilds and dominating *ESF* species with error bars indicating standard errors. Significant effects were fitted in GLMMs with negative‐binomial distributions and corrected for multiple comparisons using Tukey’s post hoc test

*OS*F species showed higher activity levels in the second period considered except for the highest canopy layer. Here, activity levels did not differ between periods (canopy gaps) or were higher in period I (forest interior; Figure 6). The *ESF* guild showed a tendency toward higher activity levels in period II with a significant increase only in the ground layer (Figure 6). For *ESF* species, we considered the dominating species separately. Barbastelle bats showed a tendency toward a higher activity in period II with no differences between height layers or canopy structures (Figure 6). Soprano pipistrelles showed activity increases in the second period for mid‐ and low‐canopy layers (Figure 6). *Myotis* species were the only group to show significantly higher activity levels in the first period considered, for all height levels in canopy gaps and for the highest canopy layer in the forest interior (Figure 6). *NSF* species revealed seasonal activity shifts depending on the canopy structure. Activity was significantly higher in period II in the forest interior but did not show seasonal variations in canopy gaps (Figure 6).

## 4 DISCUSSION

In studies dealing with the vertical stratification of temperate bat communities, sampled woodlands differ in structure and tree species, and have led to ambiguous or contrasting results even in Europe under similar climatic conditions and with comparable bat species assemblages (Froidevaux et al., 2014; Mueller et al., 2013; Plank et al., 2012). In accordance with our hypotheses, our study demonstrates differential guild‐ and species‐dependent height use by insectivorous bats in a lowland temperate old‐growth forest. We show that restricting acoustic bat sampling to the ground layer leads to a strong bias in most of the species’ activities and to wrong conclusions considering their habitat needs. Moreover, we identified a generally higher activity during the postlactation period for all species groups except *Myotis* spec. and *Myotis nattereri* and found species‐specific seasonal activity differences in height and canopy structure use.

Acoustic surveys have shortcomings that need to be addressed. We synchronously sampled canopy gaps largely void of vegetation and the forest interior, where vegetation clutter creates a completely different habitat type. This induces differences in detection probability of bat calls within the same species. However, we paid attention to place batcorders in a way that their omnidirectional ultrasonic microphones were completely surrounded by free space on a hypothetical sphere of 10 m diameter with the microphone at its center. This is important since Yates and Muzika (2006) and Bender et al. (2015) found vegetation clutter to be more important than detection probability for bat occupancy. We are therefore confident that differences in detection probability are only a minor factor influencing our results.

As expected, vertical stratification in bat activity was most pronounced in the forest interior. In forest gaps, the absence of physical constraints such as vegetation clutter caused a vertically more uniform height use pattern. Our findings corroborate studies from Adams et al. (2009) and Tiago Marques et al. (2016) who found stronger stratification in bat activity in the forest interior compared to forest edges. This edge‐interior gradient in vertical height stratification was also found for saproxylic beetles (Vodka & Cizek, 2013). Indeed, bat activity in the forest interior is mainly determined by the interaction of habitat accessibility and prey availability, whereas in open spaces such as forest gaps prey availability is the major factor driving bat activity (Adams et al., 2009; Tiago Marques et al., 2016). In contrast to Adams et al. (2009) who found generally higher activity levels in the subcanopy and canopy of the forest interior, we recorded guild‐ and species‐mediated activity patterns. Our study further indicates habitat‐dependent differences in activity levels, which highlights the role of plant species assemblages for the identification of animal–habitat relationships. This is in line with a study by Adams and Matthews (2019) on forest birds, where the influence of plant species on bird assemblages was stronger than the influence of vegetation structure. Penone et al. (2019) found that forests with a higher proportion of oak trees were more species‐rich considering forest bats than forests with a high proportion of coniferous trees. These studies show that plant species composition can integrate aspects of structural vegetation features, potential prey availability, and roosting opportunities. Therefore, plant species composition should be considered alongside structural vegetation heterogeneity when studying bat–habitat interactions.

Our results confirm that the free space above the forest canopy is used by *OSF* species (Adams et al., 2009; Kalcounis et al., 1999; Mueller et al., 2012, 2013). This habitat is especially exploited by large nyctaloids independent of the canopy structure or habitat type below them (Erasmy et al., 2021; Fukui et al., 2011). Free space within forest gaps, which is restricted in size and differs in microclimatic conditions from the aerosphere above the canopy, may represent adequate foraging habitats for smaller nyctaloids like *E*. *nilssonii* or *P. nathusii* diving into them especially at high and mid heights.

Species from the *ESF* group clearly differed in their vertical height use concurrent with results from Plank et al. (2012) and Mueller et al. (2013), while Adams et al. (2009) did not detect profound differences in stratification pattern between *ESF* species. This finding shows the limitations of the guild concept masking species‐specific habitat preferences. Barbastelle bats preferred the upper layers in the canopy gaps. Gap edge structures along tree crowns and the free space in gaps at mid and high heights probably offered highly profitable occurrences in lepidopteran prey (Rydell et al., 1996; Sierro & Arlettaz, 1997). Burford et al. (1999) and Carr et al. (2020), however, found moth species richness, occurrence, and abundance to be positively related to vegetation clutter, but Barbastelle bats—as large and rather fast flying *ESF*s—are precluded from cluttered vegetation. In the forest interior, their activity was four times lower compared to canopy gaps and restricted to the lower two layers where limited hunting possibilities were available. *P*. *pygmaeus* was virtually absent from mixed coniferous habitats, and conclusions on their height use were thus deduced from their activity recorded in broadleaved forests. In the forest interior, soprano pipistrelles were mainly active near the ground with comparable activity levels at gap ground levels. Since these bats are known to forage within the vegetation, we think that this forest layer offered the highest amount of their preferred dipteran prey (Bartonicka et al., 2008). In gaps, these bats were able to fully use the edge and open gap space over the whole vertical height spectrum, with a preference for the tree crowns as highly lucrative microhabitat. We consider the *Myotis* spec. group as consisting mostly of *Myotis brandtii* (Dietz et al., 2018; Dombrovski et al., 2017; Erasmy et al., 2021; Rachwald et al., 2001, 2021). Brandt’s bats were generally confined to the lowest layer with the highest activity levels in broadleaved gaps. Near the ground, their activity levels in gaps were nearly twice the activity levels in the forest interior. Their diet consists to a large extent of lepidopterans (Vesterinen et al., 2018), but dipterans and spiders, an indication for their gleaning foraging mode, have also been identified as main prey items (Taake, 1992). Their predominant activity at low heights in the absence of vegetation clutter in gaps may be mainly mediated by prey availability.

A similar confinement to the ground layer in gaps was found for the *NSF* guild, namely *M*. *nattereri*. In contrast to *M. brandtii*, however, *M*. *nattereri* also hunted in mixed coniferous stands. These results contrast findings by Smith and Racey (2008) and Erasmy et al. (2021) who identified a strong preference for broadleaved forests for this species, but are in accordance with Siemers et al. (1999) who found Natterer’s bats hunting indifferently in different habitat types. This contradiction might be caused by ephemeral accumulations of suitable prey in the coniferous stands. Gleaning is the main foraging strategy of Natterer’s bats (Swift & Racey, 2002). They are able to hunt close to vegetation and to very efficiently localize silent prey sitting on leaves and branches (Arlettaz, 1996; Siemers & Schnitzler, 2000; Siemers & Swift, 2006). We therefore think that Natterer’s bats in our study used low vegetation structures such as regenerating trees and low shrub vegetation in the forest interior to hunt on largely immobile prey (Siemers & Swift, 2006).

Seasonal activity patterns in temperate bats are shaped by two different mechanisms. The first mechanism is directly linked to the bats’ lifecycles. Energy demands especially of reproductive females change from pregnancy over lactation to weaning with a peak during lactation (Shiel et al., 1999). Newly volant young generally lead to an increase in the number of hunting bats in July and August (Russ et al., 2003). In early autumn, mating behavior influences nightly spatial and temporal activity patterns, while the need to accumulate fat reserves for hibernation increases the energy demands (Ciechanowski et al., 2010). Secondly, arthropod lifecycles and their temperature dependency cause variations in prey occurrences and abundances and this way influence bat seasonal activity (Höhne & Dietz, 2012; Mueller et al., 2012; Roeleke et al., 2018; Salvarina et al., 2018; Wang et al., 2010).

In concurrence with our results, Shiel et al. (1999), Russ et al. (2003), Bartonicka et al. (2008), Ciechanowski et al. (2010) and Lucan and Radil (2010) also found higher activity levels during the postlactation period. In our study, soprano pipistrelles and Brandt’s bats were the only bats with seasonal height or canopy use shifts. *P*. *pygmaeus* increased their activity during postlactation especially near the ground. Bartonicka et al. (2008) found the occurrence of certain prey groups (Neuroptera and Simulidae) to positively influence soprano pipistrelle activity increases in forest sites during postlactation. Staton and Poulton (2012) in contrary found *P*. *pygmaeus* activity during postlactation to be precluded to the forest canopy. Interspecific competition especially with the very similar *P*. *pipistrellus* is one further factor possibly influencing habitat use and therefore also height use (Davidson‐Watts et al., 2006; Roeleke et al., 2018).

For *Myotis brandtii*, higher activity levels were recorded during pregnancy and lactation, a seasonal pattern opposite to the other bat species considered. Activity peaks in gaps changed from the highest canopy layer during lactation to the ground layer during postlactation. Moreover, Brandt’s bats’ high activity levels in forest gaps during lactation decreased during postlactation. This decrease in gaps was accompanied by a slight activity increase in the forest interior. A seasonal preference for the forest interior was also found for *NSF* species. *Myotis* species are adapted to aerial hawking or gleaning in cluttered vegetation. Their activity shifts to the forest interior during postlactation could be in accordance with the arguments of Plank et al. (2012) that lactating (and postlactating) females are more agile and better able to exploit cluttered habitats than pregnant females.

## 5 CONCLUSIONS

The need to incorporate three‐dimensional structural heterogeneity in habitat–animal diversity studies has been acknowledged for different organisms (Carrasco et al., 2019; Heidrich et al., 2020; Langridge et al., 2019; Penone et al., 2019; Vodka & Cizek, 2013). Even though many studies have dealt with the role of forest structure on bat species or species groups, no clear image has yet emerged. Our study tries to complement the present picture with a focus on vertical (height in tree) and horizontal (forest interior vs. forest gaps) aspects of structural diversity. Species‐dependent differences in height and structure use become evident. Our study clearly shows that for a thorough understanding of the way bats are using forests, it is essential (i) to include the upper forest strata in the analysis, (ii) to consider seasonal changes in microhabitat use, and (iii) to focus on bat species, rather than considering bat guilds.

Recent rapid changes in European forests due to climate warming‐related stress and vitality loss will expose forest biota to enormous challenges and intensify the need for the adaptation of silvicultural concepts. In addition, wind turbines are increasingly built in Europe's forests, which will alter the space that can be exploited by forest bat communities. A thorough understanding of the interaction between bats, forest structure, and tree species composition is essential for predicting future changes in forest bat populations and bat communities and for advising related conservation efforts.

## CONFLICT OF INTEREST

The authors declare that they have no known competing financial interests or personal relationships that could have appeared to influence the work reported in this paper.

## AUTHOR CONTRIBUTION

**Maude Erasmy:** Conceptualization (lead); Formal analysis (lead); Funding acquisition (lead); Investigation (lead); Methodology (lead); Project administration (supporting); Visualization (lead); Writing‐original draft (lead); Writing‐review & editing (lead). **Christoph Leuschner:** Funding acquisition (supporting); Supervision (equal); Writing‐original draft (supporting); Writing‐review & editing (supporting). **Niko Balkenhol:** Supervision (equal); Writing‐original draft (supporting); Writing‐review & editing (supporting). **Markus Dietz:** Conceptualization (supporting); Project administration (lead); Resources (supporting); Supervision (equal); Writing‐original draft (supporting); Writing‐review & editing (supporting).

## Supporting information

Figure A1

Figure A2

Tables A1‐A3

Figure Legend

## Data Availability

Labeled raw data for all analyses are made publicly available in the Dryad Digital repository https://doi.org/10.5061/dryad.cjsxksn6m.
