# Supplementary figures and images for: Three‐dimensional stratification pattern in an old‐growth lowland forest: How does height in canopy and season influence temperate bat activity?
Source: Ecol Evol. 2021 Nov 21;11(23):17273–88. doi: 10.1002/ece3.8363 (PMC8668798; doi:10.1002/ece3.8363)

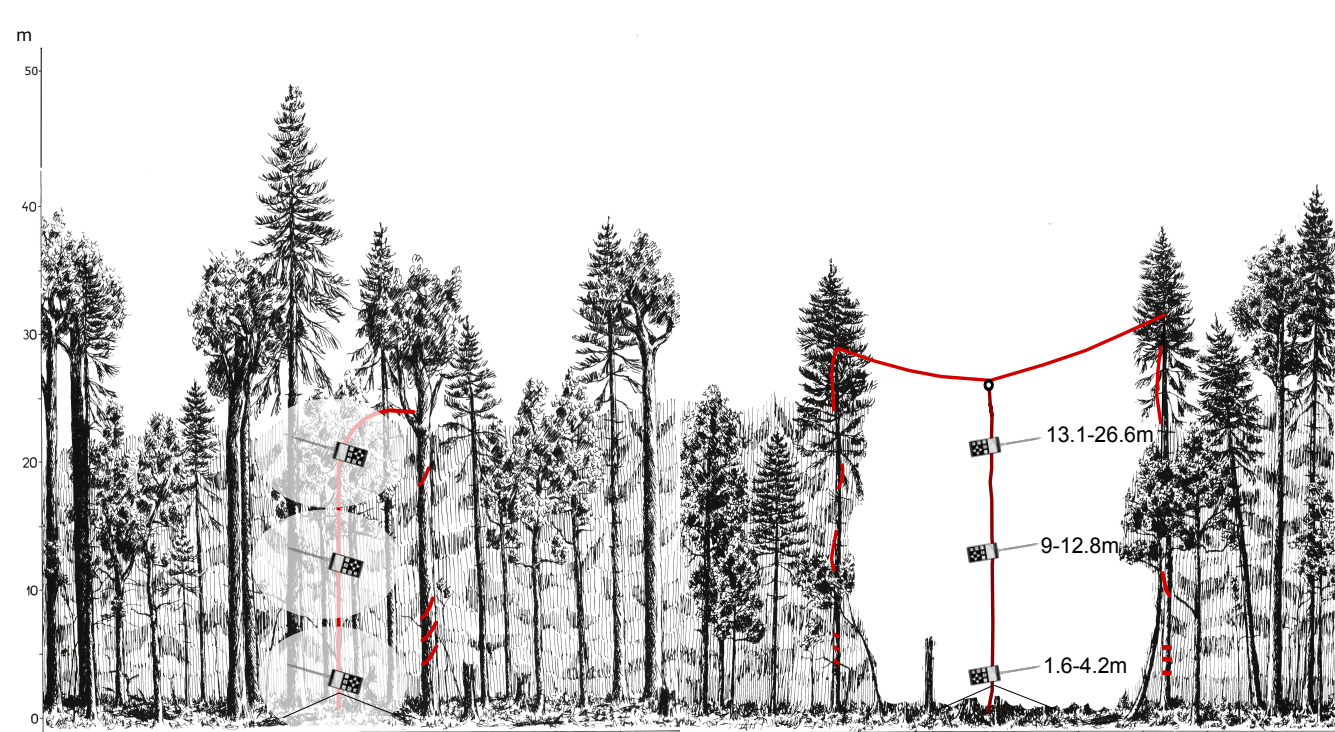

Supplement: Supplementary file 1 — Figure A1 [file ECE3-11-17273-s002.pdf]

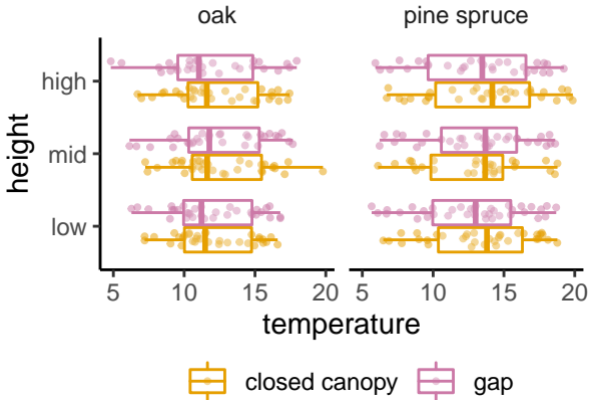

Supplement: Supplementary file 2 — Figure A2 [file ECE3-11-17273-s003.pdf]
