# Supplementary material for: Three‐dimensional stratification pattern in an old‐growth lowland forest: How does height in canopy and season influence temperate bat activity?
Source: Ecol Evol. 2021 Nov 21;11(23):17273–88. doi: 10.1002/ece3.8363 (PMC8668798; doi:10.1002/ece3.8363)
Supplement: Supplementary file 3 — Tables A1‐A3 [file ECE3-11-17273-s004.docx]

| ***habitat*** | ***structure*** | ***height*** | ***Period I*** | ***n subplots period I*** | ***Period II*** | ***N subplots period II*** |
| --- | --- | --- | --- | --- | --- | --- |
| broadleaved | Closed canopy | Low | 17 | 4 | 16 | 3 |
|  |  | Mid | 17 | 4 | 17 | 3 |
|  |  | High | 17 | 4 | 15 | 3 |
|  | Gap | Low | 17 | 4 | 16 | 3 |
|  |  | Mid | 17 | 4 | 14 | 3 |
|  |  | high | 13 | 3 | 16 | 3 |
| Mixed coniferous | Closed canopy | Low | 16 | 4 | 18 | 3 |
|  |  | Mid | 16 | 4 | 12 | 3 |
|  |  | High | 16 | 4 | 18 | 3 |
|  | Gap | Low | 16 | 4 | 21 | 3 |
|  |  | Mid | 15 | 4 | 18 | 3 |
|  |  | high | 15 | 4 | 15 | 3 |
| **SUM** | | | **192** |  | **198** |  |

table A1: Sampling effort for the two time periods considered for each habitat, canopy structure and canopy height. N subplots refers to the number of subplots sampled for each habitat category.

|  | ***Candidate model*** | ***OSF*** | ***ESF*** | ***NSF*** | ***Ppyg*** | ***Bbar*** | ***Myo*** |
| --- | --- | --- | --- | --- | --- | --- | --- |
| 1 | ~ height*period + struc + hab + mean | 12.5 | 30.5 | 5.8 | 37.9 | 9.3 | 3.4 |
| 2 | ~ height*struc + period + hab + mean | 29.7 | 8.6 | 7.8 | 14.2 | **0.8** | 8.6 |
| 3 | ~ height*hab + struc + period + mean | 14.5 | 29.4 | 8.6 | 36.2 | 9.8 | 7.7 |
| 4 | ~ structure*hab + period + height + mean | 40.7 | 38.3 | 7.6 | 50.2 | 8.9 | 8.3 |
| 5 | ~ struc*period + hab + height + mean | 39.6 | 33.3 | **0.6** | 42.3 | 3.2 | 2.9 |
| 6 | ~ height + hab + struc + period + mean | 39.2 | 37.1 | 6.5 | 48.3 | 6.8 | 6.2 |
| 7 | ~ height:period + struc:height + struc + height + period + hab + mean | 3 | **1.6** | 7.6 | **2.0** | 2.9 | 5.9 |
| 8 | ~ height:period + struc:height + struc:period + struc + height + period + hab + mean | **0** | 0 | 3.0 | 0 | 0 | 2.8 |
| 9 | ~ height:period + period:struc + period + struc + hab + mean | 13.3 | 28.5 | 0 | 55.7 | 6.1 | **0** |
|  | conditional R^2^ | 0.802 | 0.740 | 0.482 | 0.744 | 0.668 | 0.638 |
|  | marginal R^2^ | 0.543 | 0.503 | 0.266 | 0.644 | 0.405 | 0.403 |

table A2: differences in AICc-values for the candidate model sets. Final models chosen are shown in bold. Conditional and marginal R^2^-values calculated using the Nakagawa-equation from the performance package (Nakagawa and Schielzeth 2013, Lüdecke et al. 2021). All models with subplot (n=16) as random effect, and n=388 observations. Height = batcorder-position (low/mid/high), period = sampling season (I = pregnancy/lactation, II = postlactation), struc = canopy structure (gap/closed canopy), hab = habitat (broadleaved/mixed coniferous), mean = mean night-time temperature.

|  |  | ***ALL*** |  |  | ***OSF*** |  |  | ***ESF*** |  |  | ***NSF*** |  |  |
| --- | --- | --- | --- | --- | --- | --- | --- | --- | --- | --- | --- | --- | --- |
|  | **height** | **mean** | **ci** | **sum** | **mean** | **ci** | **sum** | **mean** | **ci** | **sum** | **mean** | **ci** | **sum** |
| broadleaved closed canopy | high | 1.7 | 0.9 | 54 | 0.4 | 0.4 | 14 | 1.2 | 0.6 | 39 | 0.0 | 0.1 | 1 |
|  | mid | 4.1 | 2.2 | 141 | 0.0 | 0.1 | 1 | 4.0 | 2.2 | 137 | 0.1 | 0.1 | 3 |
|  | low | 9.8 | 3.7 | 322 | 0.1 | 0.1 | 2 | 9.3 | 3.7 | 308 | 0.4 | 0.3 | 12 |
| broadleaved gap | high | 20.6 | 10.2 | 597 | 7.7 | 5.1 | 223 | 12.6 | 6.8 | 365 | 0.3 | 0.2 | 9 |
|  | mid | 10.0 | 3.6 | 309 | 0.3 | 0.2 | 9 | 9.6 | 3.6 | 297 | 0.1 | 0.1 | 3 |
|  | low | 10.4 | 2.4 | 342 | 0.2 | 0.2 | 5 | 9.1 | 2.4 | 299 | 1.2 | 0.7 | 38 |
| coniferous closed canopy | high | 1.2 | 0.5 | 40 | 0.8 | 0.4 | 28 | 0.3 | 0.2 | 10 | 0.1 | 0.1 | 2 |
|  | mid | 1.6 | 0.8 | 45 | 1.1 | 0.6 | 30 | 0.4 | 0.3 | 12 | 0.1 | 0.1 | 3 |
|  | low | 1.4 | 0.6 | 46 | 0.2 | 0.1 | 6 | 0.8 | 0.4 | 28 | 0.4 | 0.3 | 12 |
| coniferous gap | high | 6.9 | 2.0 | 206 | 5.1 | 1.6 | 153 | 1.6 | 0.7 | 47 | 0.2 | 0.2 | 6 |
|  | mid | 6.5 | 2.7 | 216 | 2.5 | 1.4 | 83 | 3.9 | 1.7 | 128 | 0.2 | 0.2 | 5 |
|  | low | 2.7 | 1.0 | 99 | 0.7 | 0.3 | 25 | 1.6 | 0.7 | 59 | 0.4 | 0.4 | 15 |
|  |  | ***Bbar*** |  |  | ***Ppyg*** |  |  | ***Myotis*** |  |  |  |  |  |
|  | **height** | **mean** | **ci** | **sum** | **mean** | **ci** | **sum** | **mean** | **ci** | **sum** |  |  |  |
| broadleaved closed canopy | high | 0 | 0 | 0 | 0.3 | 0.2 | 9 | 0.8 | 0.5 | 26 |  |  |  |
|  | mid | 1.1 | 1.0 | 37 | 1.2 | 0.8 | 42 | 1.1 | 0.6 | 36 |  |  |  |
|  | low | 0.7 | 0.5 | 22 | 4.2 | 2.5 | 139 | 2.5 | 1.1 | 84 |  |  |  |
| broadleaved gap | high | 2.1 | 1.1 | 60 | 4.7 | 1.8 | 137 | 3.8 | 3.8 | 111 |  |  |  |
|  | mid | 1.9 | 0.8 | 58 | 4.4 | 2.0 | 137 | 2.0 | 1.6 | 63 |  |  |  |
|  | low | 1.4 | 0.9 | 45 | 2.8 | 1.2 | 94 | 3.5 | 1.7 | 114 |  |  |  |
| coniferous closed canopy | high | 0.1 | 0.1 | 5 | 0.1 | 0.1 | 4 | 0.0 | 0.1 | 1 |  |  |  |
|  | mid | 0.3 | 0.2 | 7 | 0.0 | 0.1 | 1 | 0.1 | 0.1 | 3 |  |  |  |
|  | low | 0.4 | 0.3 | 13 | 0.0 | 0.0 | 0 | 0.3 | 0.2 | 10 |  |  |  |
| coniferous gap | high | 0.6 | 0.4 | 19 | 0.5 | 0.3 | 14 | 0.2 | 0.1 | 5 |  |  |  |
|  | mid | 3.0 | 1.5 | 99 | 0.4 | 0.2 | 12 | 0.3 | 0.2 | 11 |  |  |  |
|  | low | 1.1 | 0.7 | 39 | 0.1 | 0.1 | 3 | 0.4 | 0.2 | 14 |  |  |  |

table A3: Summary statistics for minute-intervals activity per night for all bat guilds and dominant ESF bat species. OSF = open-space forager, ESF = edge-space forager, NSF = narrow-space forager. Bbar = B. barbastellus, Ppyg= P. pygmaeus, Myotis = Myotis spec. except M. nattereri.
