# Supplementary material for: Three‐dimensional stratification pattern in an old‐growth lowland forest: How does height in canopy and season influence temperate bat activity?
Source: Ecol Evol. 2021 Nov 21;11(23):17273–88. doi: 10.1002/ece3.8363 (PMC8668798; doi:10.1002/ece3.8363)
Supplement: Supplementary file 4 — Figure Legend [file ECE3-11-17273-s001.docx]

Figure A1: batcorder chain setup in canopy gaps and in the forest interior, exemplary for a broadleaved plot. Figure changed after (Falinski 1986). In canopy gaps, batcorder were mostly hung up using a line spanning between two trees on each side of the gap. Batcorders were pulled up using a pulley. In the forest interior, mostly only one tree branch was necessary to pull up the batcorders attached to a string. Side strings fixed to tent pegs prevented the batcorder chain from moving around.

Figure A2: Mean nighttime temperatures for the two habitat types, two canopy structures, and for the three canopy strata sampled.
